# Preparation and Performance Evaluation of Polysaccharide–Iron Complex of *Eucommia ulmoides*

**DOI:** 10.3390/foods13142302

**Published:** 2024-07-22

**Authors:** Mengpei Liu, Yan Wang, Rong Wang, Wei Zong, Lihua Zhang, Lu Wang

**Affiliations:** 1 Key Laboratory of Cold Chain Food Processing and Safety Control, Ministry of Education, College of Food and Bioengineering, Zhengzhou University of Light Industry, Zhengzhou 450001, China; mengpei0402@163.com (M.L.); wangrong9902@163.com (R.W.); zongwei1965@163.com (W.Z.); zhanglihua82828@163.com (L.Z.); 2 State Key Laboratory of Tree Genetics and Breeding, Key Laboratory of Non-Timber Forest Germplasm Enhancement & Utilization of State Administration of Forestry and Grassland, Research Institute of Non-Timber Forestry, Chinese Academy of Forestry, Zhengzhou 450003, China

**Keywords:** *Eucommia ulmoides* leaves, physicochemical properties, structural characterization, antioxidant activity

## Abstract

An innovative iron supplement crucial for treating iron-deficiency anemia was developed in this study. Polysaccharide was extracted from *Eucommia ulmoides* leaves using a microwave-assisted hot water method, and subsequently, the polysaccharide–iron complex was synthesized through co-thermal synthesis with FeCl_3_. The physicochemical properties, structure, and thermal stability of the complex were analyzed using FE-SEM, SEC-MALLS, FT-IR, XRD, and DSC techniques. Furthermore, the antioxidant activity of the polysaccharide–iron complex was evaluated through an experiment in vitro. The results revealed that the polysaccharide–iron complex had an iron content of 6.1% and an average particle size of 860.4 nm. The microstructure analysis indicated that the polysaccharide–iron complex possessed a flaky morphology with smooth and compact surfaces. Moreover, the formation of the Fe^3+^ complex did not alter the structural framework of the polysaccharide; instead, it enhanced the polysaccharide’s thermal stability. Compared to traditional iron supplements, the *E. ulmoides*-derived polysaccharide–iron complex demonstrated significant antioxidant activity. Therefore, this novel compound exhibits significant potential as a viable iron supplement.

## 1. Introduction

According to the World Health Organization, anemia is the most prevalent form of malnutrition, affecting 33% of non-pregnant women, 40% of pregnant women, and 42% of children worldwide. Iron deficiency is identified as the primary cause. The number of anemic people worldwide reaches two billion, of which 50% can be attributed to iron deficiency. Iron-deficiency anemia can lead to various dysfunctions and ranks among the highest incidence rates for nutritional deficiencies [1]. Consequently, how to address iron deficiency anemia constitutes an urgent issue that human beings need to resolve.

At present, oral iron supplements are commonly employed for the treatment of iron-deficiency anemia. At present, iron supplements primarily consist of organic complexes, including polysaccharide–iron, heme–iron, polypeptide–iron, etc. A polysaccharide–iron complex primarily includes glucan iron, sucrose iron, and isomaltose iron [1]. Recent studies have demonstrated that polysaccharide–iron complexes not only serve as effective sources of supplemental iron but also possess the pharmacological activities of polysaccharide itself. Moreover, they offer advantages such as high iron content, good solubility, low gastrointestinal irritation, and minimal side effects. Consequently, the use of polysaccharide–iron complexes as reliable supplements has garnered increasing attention [2,3]. The plant-derived polysaccharide–iron complex is less readily available in the market and remains in the research stage. Numerous plant polysaccharides have been isolated and extracted from medicinal and edible plants, such as *Lycium barbarum* [4], *Ziziphus jujuba* [5], and *Ganoderma lucidum* [6]. In recent years, natural polysaccharides derived from plants have exhibited notable attributes, including high activity levels, low toxicity profiles, and excellent biocompatibility [7,8,9].

In addition to mouse experiments, it has been confirmed that the polysaccharide–iron complex has a positive therapeutic effect on iron-deficiency anemia [10,11,12]. It may also improve parameters associated with anemia in humans [13]. In cases of iron-deficiency anemia, a decreased activity of the immune system has been observed, prompting the combination of anti-anemia drugs with immune modulation as a potential solution [14]. The research of Wei et al. [15] confirmed that the combination of fermented silver ear polysaccharides and Fe^3+^ exhibited dual roles in iron supplementation and nutrition. The combination of polysaccharides with Fe^3+^ can feasibly achieve the dual role of supplementing iron nutrition.

*Eucommia ulmoides*, also known as Du Zhong in China, is a traditional medicinal and food resource in China. *E. ulmoides* has been utilized for over 2000 years as an effective herbal remedy for hypertension and immune enhancement. *E. ulmoides* contains various bioactive compounds, including iridoid terpenoids, lignans, phenylpropanoids, flavonoids, and polysaccharides, that possess specific functional properties [16]. Based on China’s national food safety standards, the overall safety of *E. ulmoides* leaf extract was evaluated, including genotoxicity and long-term toxicity. The research confirmed that the leaf is safe to be used as a Chinese traditional health food [17]. In this study, polysaccharide from *E. ulmoides* leaf was combined with iron in order to play the dual role of iron supplement and nutrition, providing a new perspective for a new type of iron supplement.

## 2. Materials and Methods

### 2.1. Materials

**Figure 1:**
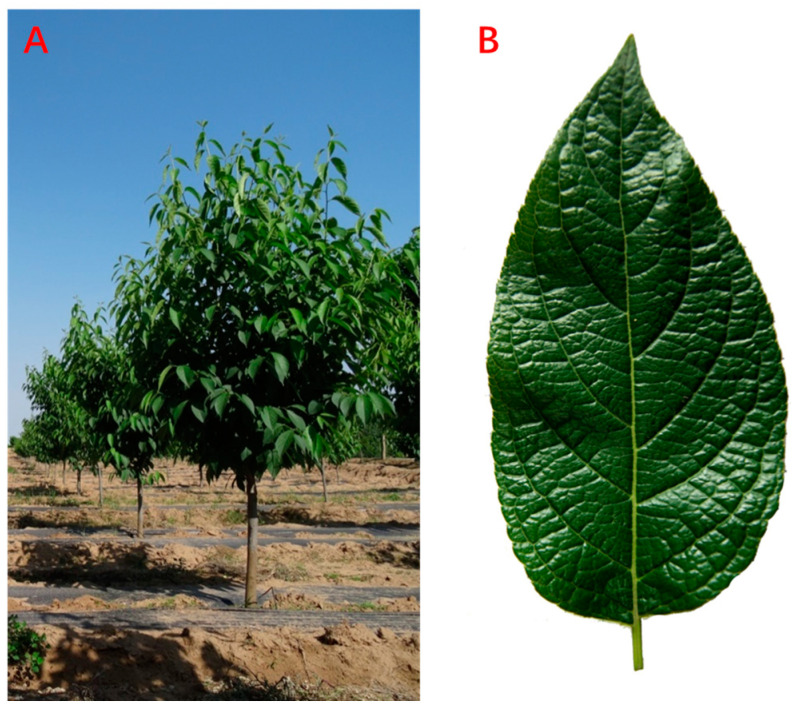
Tree and leaf shape of *E. ulmoides*: (**A**) tree shape; (**B**) leaf shape.

*E. ulmoides* leaves (500 g) were sourced from the *E. ulmoides* Research Base of Yuanyang County, Xinxiang City, Chinese Academy of Forestry, in early September 2022 (Figure 1). After harvesting, the leaves were dried at 50 °C in an oven (DHG-9030A, Yiheng Scientific Instrument Co., Ltd., Shanghai, China). Then, 50 g of dried leaves was crushed with a 60-mesh sieve for polysaccharide extraction.

### 2.2. Polysaccharide Preparation from E. ulmoides

The polysaccharide preparation from *E. ulmoides* was modified based on a previous study [18]. A precise amount of 2.0 g *E. ulmoides* leaf powder was weighed, and distilled water was used as the extraction solvent. Microwave extraction (BY-J1-3, Bayue Instrument Co., Ltd., Changsha, China) was conducted at 40 °C for 2 h with a solid–liquid ratio of 1:30, microwave time of 1.5 min, and microwave power of 230 W. After extraction, the mixture was centrifuged at 3500× *g* for 10 min, and the supernatant was concentrated at 60 °C. The concentrated liquid was mixed with anhydrous ethanol until reaching an ethanol concentration of 80%, followed by standing at 4 °C for 24 h, centrifugation, and removal of the supernatant. The crude polysaccharides of *E. ulmoides* were obtained by freeze-drying. Then, the crude polysaccharide was dissolved in distilled water and loaded onto a macroporous resin AB-8 column, eluted with distilled water first, and then eluted with an NaCl solution (0.1 mol/mL) using the DEAE-52 column chromatography method. The eluent fractions were collected automatically and monitored at a wavelength of 490 nm using the phenol–sulfuric acid method to determine its content. The obtained eluent fractions were placed into dialysis bags (molecular weight cutoff = 3500 Da) for further dialysis over a period of 24 h before being freeze-dried again to obtain *E. ulmoides* polysaccharides.

### 2.3. Preparation of Polysaccharide–Iron Complex

Referring to the method described by Jing et al. [19], 0.2 g of *E. ulmoides* polysaccharide was precisely weighed and placed into a test tube. Subsequently, 50 mg of sodium citrate and 20 mL of distilled water were added, followed by stirring in a water bath at 70 °C. Then, 0.5 mL of 10% Na_2_CO_3_ solution and 1.5 mL of 2 mol/L FeCl_3_ solution were introduced into the mixture while adjusting the pH to between 8.0–9.0 using a 20% NaOH solution. The resulting solution was stirred in a water bath at 70 °C for 1 h until the reaction reached completion, after which it was centrifuged at a speed of 7000× *g* for 4 min. The supernatant was combined with 4 times its volume of anhydrous ethanol and allowed to precipitate at—4 °C for 12 h before undergoing another round of centrifugation. The obtained precipitates were washed 3–4 times with anhydrous ethanol and subsequently freeze-dried to obtain the crude polysaccharide–iron complex. Finally, dialysis with distilled water was performed prior to preparing the polysaccharide–iron complex through freeze-drying.

### 2.4. Determination of Iron Content

The o-phenanthroline method, as described by Wang et al. [20], was employed for the determination of iron content. Initially, a standard curve for iron content was constructed using 0, 2, 3, 4, 6, 8, and 10 mL of standard iron solution in 7 separate test tubes. Subsequently, each test tube was supplemented with 1 mL of a hydroxylamine hydrochloride solution (10%), followed by the addition of 2.5 mL of an o-phenolline solution (0.1%) and finally with a glacial acetoacetic acid–sodium acetate buffer (pH = 4.5) measuring at a volume of 5 mL. The solution was diluted to a final volume of 25 mL using distilled water and incubated at a temperature of 30 °C for a duration of 15 min. Then, the absorbance was measured at a wavelength of 510 nm using an ultraviolet spectrophotometer (UV-4802, Uniko Instrument Co., Ltd., Shanghai, China). A standard curve was plotted using absorbance as the vertical coordinate and iron content (µg) as the horizontal coordinate. The regression equation was Y = 0.002X + 0.0526, and the linear correlation coefficient was R^2^ = 0.9993. Then, the iron content in the polysaccharide–iron complex was determined by the o-phenanthroline method according to the standard curve.

### 2.5. SEM Observation

The surface microstructure of polysaccharides and the polysaccharide–iron complex was examined using SEM (SU8100, Hitachi, Tokyo, Japan). The samples were coated with a thin layer of gold and observed under high-vacuum conditions at an acceleration voltage of 3 kV.

### 2.6. Average Particle Size and Zeta Potential Analysis

The polysaccharides and polysaccharide–iron complex were diluted with distilled water to prepare a solution with a concentration of 1 mg/mL. The Zeta potential and average particle size were determined at 25 °C using a Zetasizer Nano Particle Size Analyzer (ZS90, Malvern Instruments, Malvern, UK).

### 2.7. Thermal Stability Analysis

The thermal stability of the complex was assessed using differential scanning calorimetry (DSC, DSCQ20, TA Instruments, New Castle, DE, USA). First, 3 g samples were placed in an aluminum crucible; then, the test temperature range was set from 50 to 300 °C with a heating rate of 10 °C/min under nitrogen protection. The flow rate of nitrogen was maintained at 1 s/step.

### 2.8. FTIR Analysis

The polysaccharide–iron complex samples (3 mg) and KBr solid powder (300 mg) were homogenized and compressed using a tablet press. An FTIR spectrometer (Vertex 70, Bruker Instruments, Billerica, Germany) was employed to analyze the infrared absorption spectra within the range of 4000~400 cm^−1^.

### 2.9. XRD Analysis

The crystal structure of the polysaccharide–iron complex was determined using an XRD powder diffrometer (D8 Advance, Bruker Instruments, Billerica, Germany) with a diffraction angle (2ϴ) of 10° to 80°, step size of 0.05° (2ϴ), and time of 1 s/step. All XRD spectra were processed using Jade 6.0 software.

### 2.10. Molecular Weight Analysis

Referring to the method of Chen et al. [21], the molecular weight distribution of the polysaccharide–iron complex was determined. A solution containing 0.02% NaN_3_ and 50 mmol/L NaNO_3_ was prepared, followed by filtration through a 0.45 μm membrane to remove bubbles using ultrasonic treatment for 1 h. Subsequently, the complex samples were prepared as a 1 mg/mL solution and subjected to SEC-MALLS gel chromatography system (Wyatt Technology Co., Goleta, CA, USA) analysis through a 0.45 μm filter membrane to determine their molecular weight distribution. Experimental conditions included a sample size of 100 µL, a flow rate of 0.45 mL/min, and a mobile phase consisting of a mixture solution containing 0.02% NaN_3_ and 50 mmol/L NaNO_3_. Data acquisition and analysis were performed using Astra 7.0 software.

### 2.11. Antioxidant Activity In Vitro of Polysaccharide–Iron Complex

#### 2.11.1. DPPH Radical-Scavenging Activity

Based on the method proposed by Chen et al. [22], the DPPH radical-scavenging activity of the samples was determined. The polysaccharide and its iron complex samples were prepared in solution with concentrations of 0.5, 1, 1.5, 2, 2.5, and 3 mg/mL, respectively. Additionally, a DPPH solution of 0.5 mmol/L was prepared using anhydrous ethanol as the solvent. Subsequently, a mixture containing 100 µL of the sample solution and 100 µL of DPPH solution was incubated at a temperature of 37 °C for a duration of 30 min in darkness to allow for reaction completion. The absorbance A_0_ was measured at a wavelength of 517 nm using an ultraviolet spectrophotometer. The absorbance A_blank_ was obtained by replacing the DPPH solution with distilled water, while A_control_ was obtained by substituting the sample solution with anhydrous ethanol alone. Finally, the DPPH radical clearance rate of the sample was calculated according to Formula (1).

(1) $$Radical\;scavenging\;activity\left(\%\right)=\frac{{A_{control}-A}_{0}+A_{blank}}{A_{control}}\times 100$$

#### 2.11.2. OH Radical-Scavenging Activity

According to the method of Cheng et al. [23], the OH radical-scavenging activity of polysaccharide and its iron complex were determined. First, 1 mL of samples with different concentrations were added into test tubes, followed by 1 mL of 6 mmol/L ferrous sulfate and 1 mL of 6 mmol/L salicylic acid ethanol solution, and then stored at 37 °C for 15 min away from light. Then, 0.5 mL of 8.8 mmol/L H_2_O_2_ solution was added and reacted at 37 °C for 30 min. The absorbance A_0_ was measured at 510 nm by ultraviolet spectrophotometer. Distilled water was used as A_blank_ instead of the solution system, and the absorbance of distilled water instead of the sample was measured as A_control_. The OH radical-scavenging activity of the sample was calculated using Equation (1).

### 2.12. Data Processing and Analysis

Each experimental data point was repeated three times and expressed as mean ± standard error. The one-way ANOVA Tukey’s test was performed using SPSS 27 software. Origin 8.6 software was used for plotting. A significance level of *p* < 0.05 was considered statistically significant.

## 3. Results and Discussion

### 3.1. The Iron Content of Polysaccharide–Iron Complex

The iron content in the polysaccharide–iron complex was 6.1%, which was lower than that of *Enteromorpha prolifera* and *Rosa roxburghii* polysaccharide–iron complexes [24,25]. The ability of different polysaccharides to bind iron ions was different, and the reason for this difference may be related to the different structures and molecular weights of different plant polysaccharides [26].

### 3.2. Analysis of Polysaccharide–Iron Complex Microstructure

**Figure 2:**
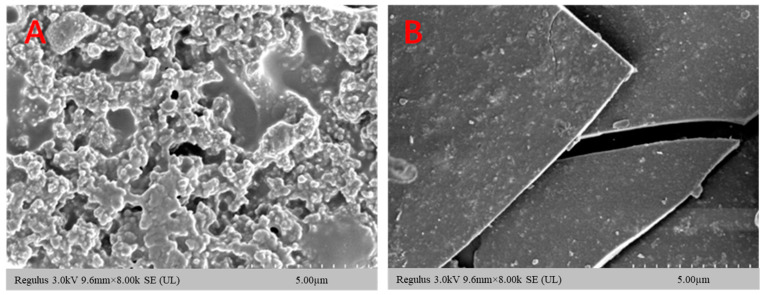
SEM of polysaccharide and its iron complex of *E. ulmoides*: (**A**) polysaccharide; (**B**) polysaccharide–iron complex.

TEM has the advantage of being more intuitive, and the microstructure of the polysaccharide–iron complex of *E. ulmoides* can be directly observed. At a magnification of 8.00 K, the microscopic morphology of the polysaccharide underwent significant changes upon combination with iron ions. The polysaccharide exhibited a loose honeycomb shape with numerous micropores on its surface, while the polysaccharide–iron complex appeared flaky with a smooth and compact surface (Figure 2). This phenomenon can be attributed to the coordination between polysaccharide and iron ions, molecular cross-linking, and conformational alterations within the polysaccharide [20,27].

### 3.3. Average Particle Size and Zeta Potential of Polysaccharide–Iron Complex

**Figure 3:**
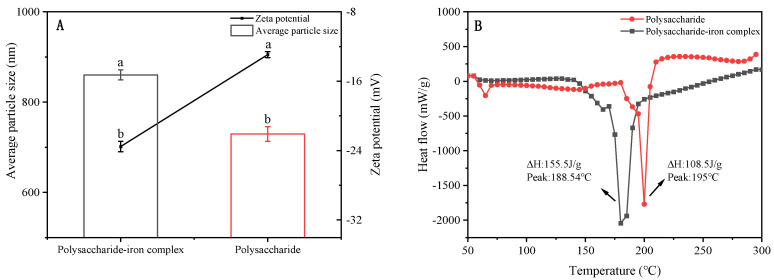
Potential particle size diagram and thermal stability of *E. ulmoides* polysaccharide and its iron complex. (**A**) Potential particle size diagram; (**B**) thermal stability; ΔH: enthalpy change. Note: Different letters indicate significant difference between different samples by Tukey’s test (*p* < 0.05). The standard error of the mean is denoted by a capped bar at the top of each column.

The average particle size of polysaccharide derived from *E. ulmoides* was measured to be 729.3 nm, while the average particle size of the polysaccharide–iron complex exhibited a significant increase to 860.4 nm. The observed phenomenon can be ascribed to the interaction between hydroxyl and carboxyl groups on the sugar chain of polysaccharides with iron during complexation, resulting in an expansion and recombination of the polysaccharide chain around the formed iron core, thereby leading to an increase in average particle size of the polysaccharide–iron complex. In addition, the Zeta potential values for both polysaccharide and its iron complex solution were −12.93 mV and −23.53 mV, respectively, indicating an increased absolute Zeta potential after complexation with iron ions (Figure 3A). The Zeta potential is directly correlated with solution stability. It has been reported that for Zeta potential values higher than 30 mV or less than −30 mV, electrostatic repulsions between particles minimize their aggregation, thereby enhancing the solution’s stability [28].

### 3.4. Thermal Stability of Polysaccharide–Iron Complex

As an excellent iron supplement, the polysaccharide–iron complex requires high stability during production, storage, and transportation. Insufficient stability may lead to structural instability and compromise the desired effect of iron supplementation. The results revealed that the endothermic reaction took place within the temperature range of 150–200 °C, wherein the peak denaturation temperature and enthalpy change of polysaccharides were determined to be 195 °C and 108.5 J/g, respectively. Meanwhile, for the polysaccharide–iron complex, the corresponding peak temperature and enthalpy change were found to be 188.54 °C and 155.5 J/g, respectively. Compared with polysaccharides, the denaturation peak temperature of the polysaccharide–iron complex did not change significantly, but the enthalpy change increased (Figure 3B). Therefore, the polysaccharide–iron complex had higher thermal stability than polysaccharides. On the one hand, this phenomenon may be attributed to the complexation of polysaccharides with iron ions, facilitating the ordered arrangement of polysaccharide molecules and enhancing the stability of the complex. This finding was consistent with previous studies on iron complexes of *Astragalus membranaceus* polysaccharide, corn silk polysaccharide, and Spirulina polysaccharide [29,30]. On the other hand, Fe^3+^ can potentially chelate with polar groups on the polysaccharide to form a stable β-FeOOH iron core, thereby increasing its spatial structural stability and improving its thermal stability.

### 3.5. Infrared Spectrum Analysis of Polysaccharide–Iron Complex

**Figure 4:**
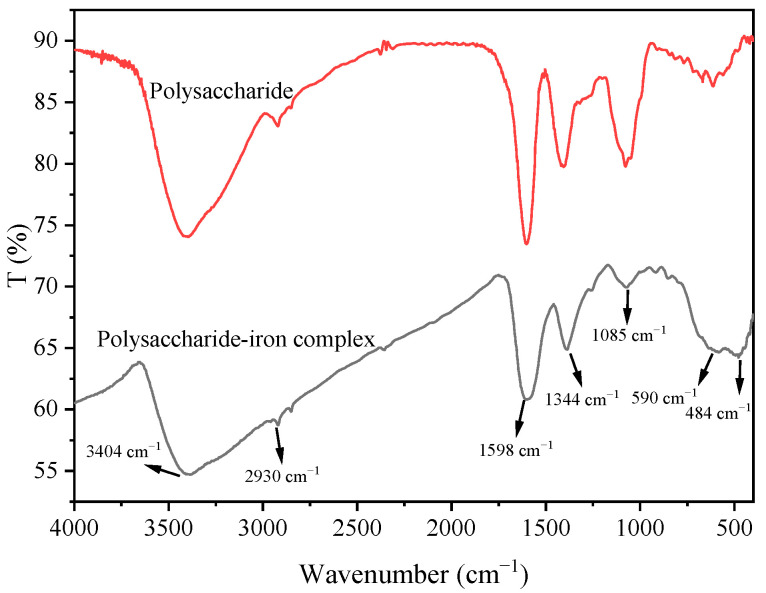
FTIR of *E. ulmoides* polysaccharide and its iron complex.

FTIR is a crucial tool for the analysis of functional groups, offering simplicity and selectivity for qualitative compound analysis [31]. The broad absorption peak at 3404 cm^−1^ corresponded to hydroxyl group stretching vibrations, while the weak absorption peak at 2930 cm^−1^ represented C-H bond stretching vibrations. At 1598 cm^−1^ and 1344 cm^−1^, these peaks corresponded to C=O tensile vibrations and O-H bending vibrations, respectively. The absorption peak at 1085 cm^−1^ arose from C-O stretching vibrations. The infrared spectrum of the polysaccharide–iron complex of *E. ulmoides* was similar to the polysaccharide sample. However, compared to the polysaccharide sample, the peaks associated with the polysaccharide–iron complex at 3404 cm^−1^, 1598 cm^−1^, 1344 cm^−1^, and 1085 cm^−1^ significantly weakened, indicating that the binding process primarily affected hydroxyl and carboxyl groups within polysaccharides [32]. Additionally, a new absorption peak near 484 cm^−1^ emerged in the polysaccharide–iron complex. Literature reports indicate that the observed peaks at approximately 438 cm^−1^ and 570 cm^−1^ predominantly correspond to characteristic Fe-O bonds in iron oxide, suggesting the presence of a β-FeOOH polymerized iron core structure [33] (Figure 4). These findings demonstrated that the complexation of polysaccharide with Fe^3+^ did not change the structural framework of polysaccharide, but C-H and C-O bonds in polysaccharide participated in the complexation, leading to a reduction in the corresponding absorption peak intensity.

### 3.6. XRD Analysis of Polysaccharide–Iron Complex

**Figure 5:**
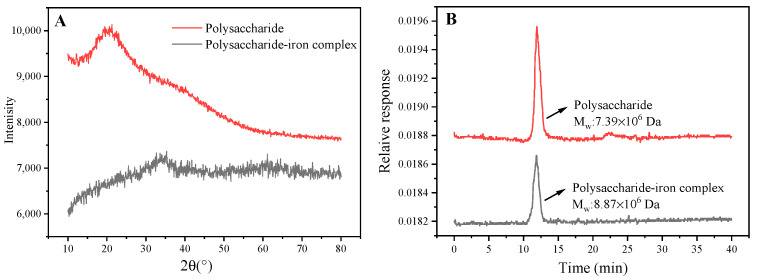
XRD pattern and molecular weight (Mw) of *E. ulmoides* polysaccharide and its iron complex. (**A**) XRD; (**B**) Mw.

XRD is a crucial analytical technique for investigating the crystal structure of polysaccharide. Due to variations in chemical composition and structural parameters among different substances, distinct diffraction patterns are generated when X-rays pass through crystals, serving as key indicators for identifying substance structures [34]. The diffraction pattern of polysaccharide exhibited a broad peak at 20°, suggesting its amorphous nature. In contrast to polysaccharide, the polysaccharide–iron complex peak disappeared at 20°, which may be due to weakened hydrogen bond interactions resulting from Fe^3+^ introduction [35]. The dispersion peak of the polysaccharide–iron complex exhibited a “steamed bun” shape with a widened pattern and no sharp absorption peaks, indicating that the fundamental skeleton of the polysaccharide remained intact. This finding was consistent with the results obtained from infrared spectra analysis. Moreover, new diffraction peaks emerged at 30° and 60° in the polysaccharide–iron complex (Figure 5A). According to the study conducted by Zhang et al. [36], this provided further evidence for the formation of novel compounds resulting from reactions between polysaccharide and Fe^3+^.

### 3.7. Molecular Weight of Polysaccharide–Iron Complex

As illustrated, the molecular weight of polysaccharide was measured at 7.387 × 10^6^ Da, while the polysaccharide–iron complex exhibited a higher molecular weight of 8.869 × 10^6^ Da, indicating a significant increase upon binding with iron ions (Figure 5B). These findings were consistent with previous studies on the iron complexation of *Enteromorpha prolifera* and *Astragalus membranaceus* polysaccharide [24,29]. Additionally, a previous study found that *E. ulmoides* polysaccharides were mainly composed of L-rhamnose, D-arabinose, D-mannose, D-glucose, D-galactose, and D-xylose, among which D-arabinose and D-galactose had the highest content, and D-xylose had the lowest [37].

### 3.8. Antioxidant Activity Analysis of Polysaccharide–Iron Complex

**Figure 6:**
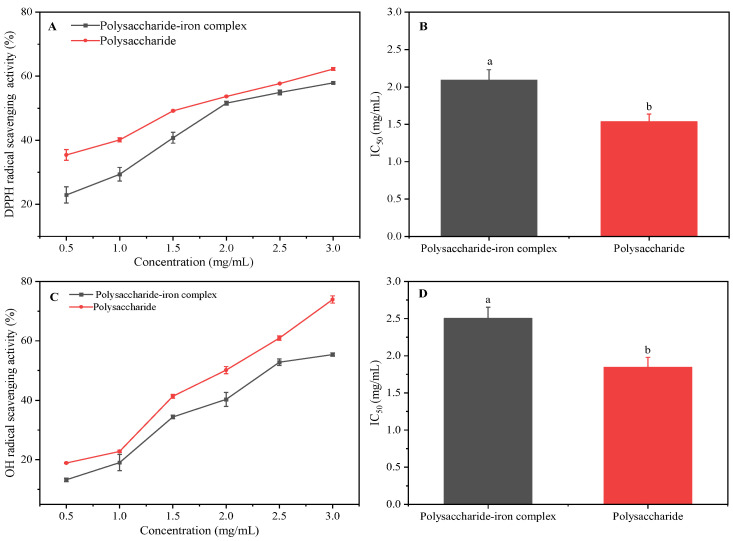
Radical-scavenging activity of *E. ulmoides* polysaccharide and its iron complex. (**A**) DPPH radical-scavenging rate; (**B**) IC_50_ of DPPH radical-scavenging activity; (**C**) OH radical-scavenging rate; (**D**) IC_50_ of OH radical-scavenging activity. Note: Different letters indicate significant difference between different samples by Tukey’s test (*p* < 0.05). The standard error of the mean is denoted by a capped bar at the top of each column.

DPPH and OH radical activities were used to detect the antioxidant activity of the *E. ulmoides* polysaccharide–iron complex. The results demonstrated that within the concentration range of 0.5–3.0 mg/mL, there was a noticeable dose-dependent effect as the sample concentration increased, leading to an enhanced DPPH scavenging ability of both polysaccharide and the iron complex. At a sample concentration of 3.0 mg/mL, the scavenging rate for the polysaccharide reached 62%, while the scavenging rate for the polysaccharide–iron complex was determined to be 58%. Correspondingly, their IC_50_ values were calculated as 1.54 mg/mL and 2.09 mg/mL, respectively (Figure 6A,B). These findings indicated that compared to *E. ulmoides* polysaccharide alone, the DPPH scavenging ability of the polysaccharide–iron complex exhibited a relatively lower efficacy. Moreover, the results showed that in the concentration range of 0.5–3.0 mg/mL, the OH radical-scavenging activity of *E. ulmoides* polysaccharide and its complex was significantly dose-dependent. At 3.0 mg/mL, the radical-scavenging rate of polysaccharide and the polysaccharide–iron complex was 74% and 55%, and the IC_50_ value was 1.85 mg/mL and 2.50 mg/mL, respectively (Figure 6C,D). Similarly, the OH radical-scavenging activity of the polysaccharide–iron complex was lower than that of polysaccharide.

The results of the antioxidant activity demonstrated that the polysaccharide–iron complex of *E. ulmoides* exhibited a certain level of radical-scavenging activity, lower than that of the polysaccharide alone. This finding was consistent with the study of Yu et al. [38]. The formation of a stable “β-FeOOH” iron core structure during the binding process between polysaccharide and iron hindered the efficient transfer of H protons in polysaccharide, consequently reducing its antioxidant activity. Moreover, in the process of scavenging radicals, polysaccharides could act as hydrogen atom or electron donors, with hydroxyl and carboxyl groups being major contributors. The complexation between polysaccharide and iron diminished its ability to provide electrons, thereby compromising its antioxidant capacity.

## 4. Conclusions

The polysaccharide–iron complex of *E. ulmoides* was synthesized through the co-thermal synthesis of FeCl_3_, and its physicochemical properties, structural characterization, thermal stability, and antioxidant activity were investigated in this study. The results demonstrated that the polysaccharide–iron complex had an iron content of 6.1%. The average particle size of the complex was measured at 860.4 nm with an absolute Zeta potential of 23.53 mV. Microscopically, the polysaccharide–iron complex displayed a flaky structure with a smooth and compact surface morphology. The peak temperature and enthalpy change for the polysaccharide–iron complex were determined to be 188.54 °C and 155.5 J/g, respectively. Additionally, infrared spectroscopy revealed that binding with Fe^3+^ did not alter the basic structure of polysaccharide. XRD analysis confirmed characteristic absorption peaks at 30° and 60° associated with iron ions within the composite material while also indicating an increase in molecular weight. Furthermore, the antioxidant activity of the polysaccharide–iron complex of *E. ulmoides* showed IC_50_ values for DPPH scavenging (2.09 mg/mL) and OH scavenging (2.50 mg/mL), which were slightly lower than those observed for polysaccharide alone. However, compared with the traditional iron supplement, the polysaccharide–iron complex of *E. ulmoides* has certain antioxidant activity. The results of this study will provide a reference for the development of the polysaccharide–iron complex of *E. ulmoides* as a new type of iron supplement.

However, there are still some shortcomings in this study. The physicochemical properties of the *E. ulmoides* polysaccharide–iron complex need to be further studied. In addition to the in vitro antioxidant capacity, the in vivo antioxidant capacity of the complex needs to be further verified. Moreover, *E. ulmoides* polysaccharides also have anti-inflammatory and immune-enhancing effects, and the pharmacological activity is also worth studying. Further, the biological activity of the complex is closely related to its structure, so the structure–activity relationship of the polysaccharide–iron complex is noteworthy. At present, the polysaccharide–iron complex is still in the research and development stage, and relatively few products are on the market. Therefore, it is necessary to further study the polysaccharide–iron complex to provide health support for the iron-deficiency anemia population.

## Data Availability

The original contributions presented in the study are included in the article; further inquiries can be directed to the corresponding author.
